# From Asia to Europe: epidemiology, genetic diversity, and One Health implications of *Thelazia callipaeda*

**DOI:** 10.1051/parasite/2026032

**Published:** 2026-06-03

**Authors:** Piyanan Taweethavonsawat, Napassarawadee Tramas, Duriyang Narapakdeesakul, Sariya Asawakarn, Frederic Beugnet

**Affiliations:** 1 Veterinary Parasitology Unit, Department of Pathology, Faculty of Veterinary Science, Chulalongkorn University Bangkok 10330 Thailand; 2 Biomarkers in Animals Parasitology Research Unit, Faculty of Veterinary Science, Chulalongkorn University Bangkok 10330 Thailand; 3 The International Graduate Program of Veterinary Science and Technology (VST), Faculty of Veterinary Science, Chulalongkorn University Bangkok 10330 Thailand; 4 Biochemistry Unit, Department of Veterinary Physiology, Faculty of Veterinary Science, Chulalongkorn University Bangkok 10330 Thailand; 5 Boehringer Ingelheim Animal Health, 29 Av. Tony Garnier, Lyon, 69007, France / Veterinary Parasitology Unit, Department of Pathology, Faculty of Veterinary Science, Chulalongkorn University Bangkok 10330 Thailand

**Keywords:** Ocular parasitism, One Health, *Thelazia callipaeda*, Thelaziosis, Zoonotic nematode

## Abstract

*Thelazia callipaeda*, commonly known as the oriental eyeworm, is a vector-borne parasitic nematode that infects the ocular tissues of a wide range of mammalian hosts, including dogs, cats, wildlife, and humans. Historically confined to East and Southeast Asia, *T. callipaeda* has emerged over the past 2 decades as a significant zoonotic parasite in Europe, with an expanding geographic distribution driven by the spread of lachryphagous drosophilid fruit fly vectors of the genus *Phortica*. This review synthesizes current knowledge on the taxonomy, epidemiology, biology, genetic diversity, pathogenesis, and control of *T. callipaeda*, with particular emphasis on its One Health relevance. Molecular studies reveal low but structured genetic variability, characterised by a single predominant haplotype circulating in Europe and high haplotype diversity in Asian populations, reflecting long-term endemicity and distinct transmission dynamics. Clinically, infection can result in ocular irritation ranging from mild conjunctivitis to severe keratitis and corneal ulceration, with dogs acting as the primary domestic reservoir and wildlife sustaining sylvatic transmission cycles. Human infections, though underreported, are increasingly recognised and pose a growing public health concern. Effective management relies on mechanical worm removal, macrocyclic lactone treatment and prevention, and integrated surveillance of animal hosts and vectors. Given the influence of climate change, animal mobility, and environmental factors on vector ecology, coordinated One Health strategies are essential to mitigate the continued spread and zoonotic impact of this emerging eyeworm.

## Abstract (fr)

*Thelazia callipaeda*, communément appelé ver oculaire oriental, est un nématode parasite transmis par vecteur qui infecte les tissus oculaires d'une grande variété d'hôtes mammifères, notamment les chiens, les chats, la faune sauvage et les humains. Historiquement confiné à l'Asie de l'Est et du Sud-Est, *T. callipaeda* est devenu, au cours des deux dernières décennies, un parasite zoonotique important en Europe, avec une aire de répartition géographique en expansion, favorisée par la propagation des mouches des fruits lacryphages du genre *Phortica*. Cette revue synthétise les connaissances actuelles sur la taxonomie, l'épidémiologie, la biologie, la diversité génétique, la pathogénie et la lutte contre *T. callipaeda*, en mettant l'accent sur son importance dans le cadre de l'approche « Une Seule Santé ». Les études moléculaires révèlent une variabilité génétique faible mais structurée, caractérisée par un haplotype prédominant unique en Europe et une forte diversité d'haplotypes dans les populations asiatiques, témoignant d'une endémicité de longue date et de dynamiques de transmission distinctes. Cliniquement, l'infection peut provoquer une irritation oculaire allant d'une conjonctivite légère à une kératite sévère et à un ulcère cornéen. Les chiens constituent le principal réservoir domestique et la faune sauvage assure la transmission sylvatique. Bien que sous-déclarées, les infections humaines sont de plus en plus souvent diagnostiquées et représentent un problème de santé publique croissant. Une prise en charge efficace repose sur l'extraction mécanique des vers, le traitement et la prévention par lactones macrocycliques, ainsi que sur une surveillance intégrée des hôtes et vecteurs animaux. Compte tenu de l'influence du changement climatique, de la mobilité animale et des facteurs environnementaux sur l'écologie vectorielle, des stratégies « Une Seule Santé » coordonnées sont essentielles pour limiter la propagation continue et l'impact zoonotique de ce ver oculaire émergent.

## Introduction

*Thelazia callipaeda* Railliet and Henry, 1910 [17, 43, 44] is a parasitic nematode classified within the phylum Nematoda, and class Chromadorea. It belongs to the order Spirurida, within the superfamily Thelazioidea, and is part of the family Thelaziidae, genus *Thelazia*.

Thelaziidae are commonly known as “eyeworms”; these parasites primarily inhabit the conjunctival sacs, lacrimal glands, or tear ducts of various vertebrates, including humans [44]. They are thin, thread-like, whitish worms. Females typically measure 12–20 mm, while males are smaller, around 5–12 mm. Their cuticle features transverse striations, giving them a wrinkled appearance, and playing a role in their pathogenicity by causing irritation [43, 44]. While considered endoparasites, they live in areas exposed to the external environment, specifically the surface of the eye, conjunctival sacs, and lachrymal ducts. This location allows for an indirect life cycle requiring intermediate hosts, typically non-biting flies from the families Drosophilidae (fruit flies) or Muscidae (little house flies). Flies become infected by feeding on the ocular secretions of an infected host, which contain first-stage larvae (L1). Inside the fly, the larvae develop into the infective third stage (L3) over approximately 2–3 weeks. The fly deposits the L3 larvae back onto the eye of a new definitive host during subsequent feeding. The worms reach the adult stage in about a month [3, 41]. The family Thelaziidae includes 7 genera, with *Thelazia* being the most medically and economically significant [44]. The main *Thelazia* species are: *Thelazia callipaeda* (Oriental eyeworm), the most widespread species, infecting dogs, cats, foxes, and humans, primarily across Asia and Europe; *Thelazia californiensis*, found mainly in the western United States (*i.e.* California and New Mexico), affecting dogs, deer, and occasionally humans; *Thelazia gulosa* and *Thelazia skrjabini*, primarily infecting ruminants, with global distribution; and *Thelazia lacrymalis*, primarily infecting horses, with global distribution [44].

Regarding the two species infecting dogs, *T. callipaeda* and *T. californiensis*, the first appears to originate from Asia, transmitted by fruit flies of the genus *Phortica*, and spreading globally [17], while the second seems to be restricted to the western coast of North America and is transmitted by small house flies of the genus *Fannia* [44, 53]. Nevertheless, complete molecular analyses of *T. californiensis* are not available to clearly demonstrate that it is an independent species, or to relate it to the other species of the genus [45, 55].

Infestation by these worms causes several symptoms including conjunctivitis, excessive tearing (epiphora), and foreign body sensation with itching (pruritus). In severe or untreated chronic cases, corneal ulcers or visual impairment can occur. The primary treatment is mechanical removal of the worms using forceps or saline solution flushing. Prevention is possible in endemic areas through regular use of macrocyclic lactone formulations [27].

*Thelazia callipaeda* is commonly referred to as the “oriental eyeworm” as it has widespread presence throughout numerous parts of China and Southeast Asia (*i.e.* Thailand, Malaysia, Indonesia, and the Philippines) [5, 43] and is the primary causative agent of ocular thelaziosis in humans and animals. The first description was made by Railliet and Henry in 1910, two French parasitologists. The mature female worm first described was sent to them by a military veterinarian (*i.e*. Lieutenant Dale) who extracted several worms from a dog in Rawalpindi (south of Islamabad, Pakistan, formerly in the province of Punjab) [14, 42, 44].

## Epidemiology

**Figure 1:**
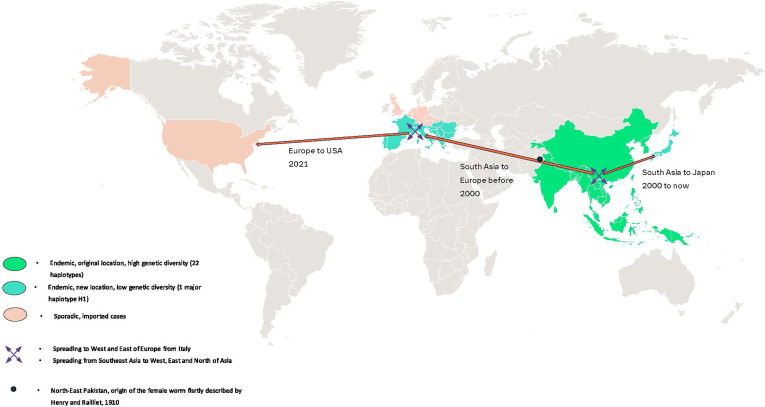
Geographical distribution and extension of *Thelazia callipaeda*, the oriental eyeworm.

Over the past two decades, the geographic range of *T. callipaeda* has expanded considerably (Fig. 1). Once restricted to East and Southeast Asia (originally described in Southern China and Thailand), the parasite spread north and west throughout Asia and was first imported into Italy. The first European cases were described in northern Italy (Piedmont) by Rossi *et al.* (1989) [49], but it appears that foci were already present in southern Italy (Basilicata region) [40, 42, 48].

The species is now established across Europe, with increasing reports in many countries, including Austria, Croatia, France, Germany, Greece, Italy, Moldova, Portugal, Romania, Slovakia, Spain, and Switzerland (alphabetical order) [8, 9, 15, 20, 33–36, 47, 50–52, 60]. Researchers believe the parasite first arrived in Italy from Asia, where it was described in 1989 [50], then spread north and west to Switzerland, France and Spain, followed by eastern European countries along the Adriatic Sea [34]. Since then, the spread has accelerated, continuously reaching new regions across Europe. Sporadic cases have recently appeared in the United States of America, especially in the New York area, typically linked to imported animals [29, 53, 54]. In Asia, *T. callipaeda* has spread from the south to the north and has been described sporadically in Japan from 1957 (first case in the South of Japan) to the year 2000 [10]. Since 2000, it has been spreading in Japan, where it is now endemic in wild carnivores [12, 21, 25, 56, 57].

Amongst several factors including fruit agriculture, the expansion of fruit fly vectors belonging to the genus *Phortica* drives the spread in Europe [19, 20, 24, 37]. Transmission occurs when male *Phortica* flies feed on the lacrimal secretions of mammals [37]. The principal European vector is *Phortica variegata* [2, 4, 37, 43, 44, 49, 62], while in East Asia it is *Phortica okadai* [24]. Other drosophilid species like *Phortica oldenbergi* may also act as vectors [2, 4]. As a result, infection risk is highly seasonal [37, 49], peaking during periods of vector activity (i.e. late spring through early autumn in temperate regions, and nearly year-round in tropical climates such as Thailand).

*Thelazia callipaeda* infects a broad host spectrum, including dogs, cats, foxes, wolves, martens, bears, lagomorphs, and humans [[5, 12, 18, 23–25, 40, 41, 46, 57]. Domestic and wild canids represent the primary reservoir and the species most frequently affected, corresponding to the high prevalence observed in canine populations across both Asia and Europe. Wild carnivores sustain sylvatic transmission cycles, ensuring persistence even in areas with strict veterinary control measures.

Prevalence varies considerably by region. The main reservoir remains wild carnivores, especially wild canids, but also other mammals such as rabbits [5, 12, 18, 24, 25, 40, 41, 46, 57]. In endemic Asian countries, canine infection proportions can reach over 84.6% [10, 56], while in Europe, prevalence has risen since the early 2000s. In a study by Miró *et al.* (2011) [35], focusing on a Spanish hotspot, *T. callipaeda* was detected in 182 dogs, representing 39.9% of the population examined. Factors driving this expansion include agricultural activities (*i.e.* fruit production), increased human and animal mobility, favourable climatic conditions for vectors, and heightened diagnostic awareness. Human thelaziosis, though relatively rare, is being reported with increasing frequency, predominantly in Asia but also in Europe [6, 7, 13, 21, 30, 32, 34, 39, 42, 48, 61]. Children and the elderly are particularly susceptible due to outdoor exposure, reduced hygiene, or close contact with infected animals. The expanding distribution, zoonotic potential, and associated morbidity underscore the parasite’s growing public health significance [14, 42, 44].

In summary, the epidemiology of *T. callipaeda* reflects a complex interaction between expanding vector populations, mobile reservoir hosts, and climatic conditions beneficial to fruit fly activity. Ongoing surveillance, molecular characterisation of parasite strains, and ecological studies of vectors are essential for effective monitoring and control of this emerging zoonotic eyeworm.

## Biology

**Figure 2:**
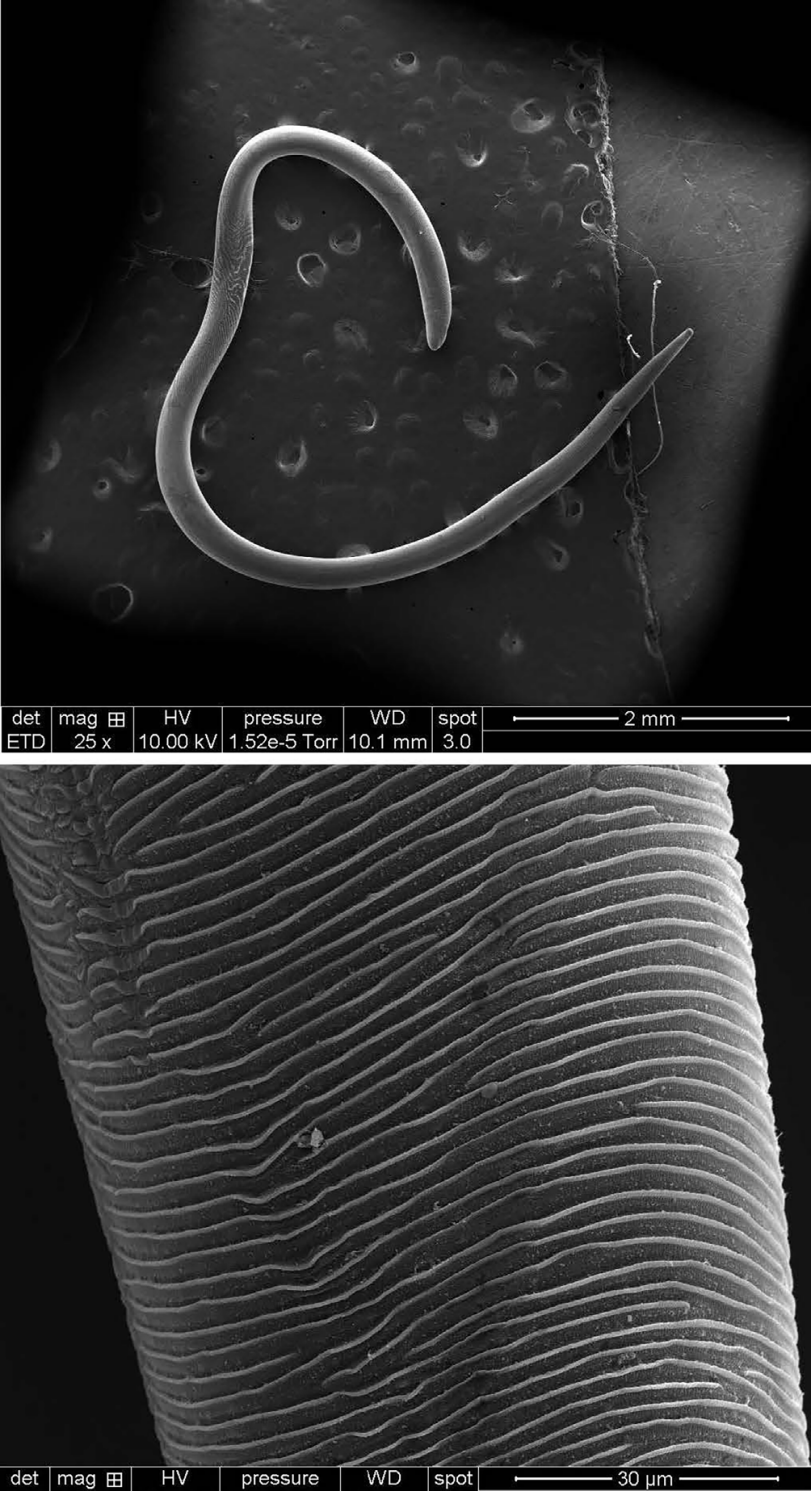
Scanning electron micrographs of *Thelazia callipaeda* showing (a) the slender, thread-like adult worm, (b) fine transverse cuticular striations.

**Figure 3:**
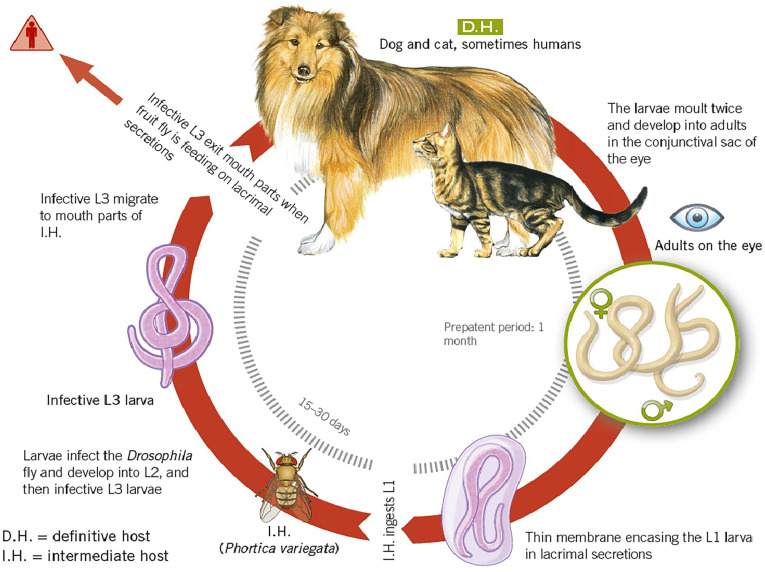
*Thelazia callipaeda* life cycle. From Beugnet *et al.* Textbook of Clinical Parasitology in dogs and cats. 2025, 436 p. ISBN: 978-2-9593929-0-0.

*Thelazia callipaeda* is a spirurid nematode that parasitises the ocular tissues of mammals, most notably the conjunctival sacs and lacrimal ducts of the eye [3, 14, 43, 45]. Adult worms are slender, whitish, and threadlike (Fig. 2), with marked sexual dimorphism: females reach approximately 13–17 mm in length, while males are smaller at 9–13 mm [45, 62]. Their cuticle bears fine transverse striations (Fig. 2), and they possess a distinctive buccal capsule that enables firm attachment to the conjunctival mucosa [30]. In addition, *T. callipaeda* does not colonise internal organs. The indirect, vector-borne life cycle of *T. callipaeda* relies on drosophilid fruit flies of the genus *Phortica* as intermediate hosts (Fig. 3) [3, 37, 44, 45]. Reproduction is ovoviviparous – females retain eggs until hatching, releasing first-stage larvae (L1) directly into the tear film. Male *Phortica* flies ingest these larvae during lachryphagy, and within the digestive tract of the fly, the L1 larvae develop into infective third-stage larvae (L3) over approximately 35 days [37, 45, 62], depending on environmental conditions. This maturation occurs in the digestive and reproductive organs. Once fully developed, L3 larvae migrate to the mouthparts of the fly, enabling transmission to a new host during subsequent feeding on tears of the animal.

The process is strongly seasonal and closely linked to the activity of *Phortica* species [49, 62]. A multicentre survey conducted in Spain, Italy, and New Jersey (USA) has demonstrated that the main factor for fly activity in *P. variegata* in temperate countries is temperature related, with a peak of vector density between May and September, depending on the geographical location and the year, when daily temperatures are comprised between 20 °C and 30 °C [49].

The biology of *T. callipaeda* demonstrates a remarkable specialisation for survival on the ocular surface of mammals and a close ecological dependence on its drosophilid vectors. This unique adaptation reinforces its ability to infect a diverse range of hosts and highlights its growing importance as an emerging zoonotic pathogen [14, 42].

## Genetic diversity

Since the turn of the century, molecular investigations of *T. callipaeda* have been exclusively conducted in Asia, Europe, and the Americas [9, 12, 14, 20, 21, 23, 25, 30, 56, 64, 65]. Through the analysis of mitochondrial cytochrome *c* oxidase subunit 1 (*cox*1) sequences utilising our dataset (114 reference sequences from GenBank database, 613-bp excluding gaps) (Table 1), at the global scale, this parasite demonstrates an average GC content of 34.9%, with 29 variable sites (VS) and 22 distinct haplotypes (H) identified. The average haplotype (Hd) and nucleotide (*π*) diversities were calculated to be 0.839 and 0.01305, respectively. Naturally, *T. callipaeda* exhibits the greatest levels of genetic diversity in its ancestral region in Asia, with at least 21 distinct haplotypes (H1–H21) detected. Within this region, the Chinese population has the greatest haplotype diversity (H = 18, Hd = 0.908), while populations in Japan show a comparatively lower diversity with only two haplotypes identified (H = 2, Hd = 0.517). In contrast, populations from Europe and North America show no polymorphism within this dataset. All sequences, except one, analysed from these continents belonged to a single, identical haplotype (H1) [9], yielding in diversity indices of zero across 13 European countries and the USA. A recent European isolate from a human case was identified as H22 [9].

**Table 1:** Polymorphism and genetic diversity of *T. callipaeda cox*1 sequences discovered across various countries, continents, and hosts globally, based on the *cox1* 613-bp alignment dataset.

Origin	*N*	GC%	VS	*H*	Hd (SD)	*π* (SD)	Lists of GenBank accession numbers utilised for analysis
**Country**							
China	47	34.4	19	18	0.908 (0.023)	0.00688 (0.00056)	KY908318–KY908320, MF795663–MF795694, MN719909, MN719911–MN719914, MT040339–MT040344, NC018363
Japan	26	34.7	14	2	0.517 (0.031)	0.01181 (0.00072)	AB852543–AB852550, AB538283, LC790039, LC746896–LC746898, LC818871–LC818880, PP094558, PV291673–PV291674
India	1	35.7	0	1	0	0	PX482520
Romania	8	35.6	0	1*	0	0	KT716012–KT716013, KP087796, MN176281–MN176282, MH622760, OQ298931, PQ600880, PX503837
Hungary	7	35.6	0	1*	0	0	PX498019–PX498025
Slovakia	7	35.6	0	1*	0	0	KY476400, MF155930, MF578281, MK546436–MK546439
Portugal	3	35.6	0	1*	0	0	KX033489, OM327770, OM470911
Serbia	3	35.6	0	1*	0	0	KJ433982–KJ433983, OP696980
Estonia	2	35.6	0	1*	0	0	PX498064–PX498065
Greece	2	35.6	0	1*	0	0	MG913802, OK662943
Italy	2	35.6	0	1*	0	0	OM462655, ON713991
Moldova	1	35.6	0	1*	0	0	MN163032
Spain	1	35.6	0	1*	0	0	PX381501
Austria	1	35.6	0	1*	0	0	PQ600880
USA	3	35.6	0	1*	0	0	MW570733, OR982681, PP739308
**Worldwide**	114	34.9	29	22	0.839 (0.026)	0.01305 (0.00049)	Accession numbers from 15 aforementioned countries
**Continent**							
Asia	74	34.5	29	21	0.905 (0.016)	0.01069 (0.00076)	Accession numbers from China, Japan, and India
Europe	37	35.6	0	1*	0	0	Accession numbers from Romania, Hungary, Slovakia, Portugal, Serbia, Estonia, Greece, Italy, Moldova, Spain, and Austria
Americas	3	35.6	0	1*	0	0	Reference sequences from the USA
**Host**							
Humans	42	34.5	27	14	0.897 (0.026)	0.00963 (0.00111)	AB538283, KY908318–KY908320, LC790039, MF795663–MF795694, OP696980, OM327770, OM470911, PP094558, PQ600880
Canidae	47	35.1	22	9	0.693 (0.064)	0.01206 (0.00117)	AB852543–AB852548, AB852550, KJ433982, KP087796, KT716012–KT716013, KY476400, LC818874–LC818875, LC818878–LC818879, LC781880, LC746896, MF578281, MF155930, MG913802, MK546436–MK546439, MN163032, MN719909, MT040339–MT040344, MW570733, NC018363, OM462655, PX482520, PX498022–PX498025, PX498019–PX498021, PX498064–PX498065
Felidae	7	35.2	14	4	0.714 (0.181)	0.00994 (0.00275)	AB852549, KJ433983, MH622760, MN719911, MN719913, OQ298931, OR982681
Mustelidae	6	35.2	21	3	0.733 (0.155)	0.01740 (0.00356)	LC818872, MN176281–MN176282, PV291673–PV291674, PX381501
Ursidae	6	35.3	21	4	0.800 (0.172)	0.01381 (0.00482)	LC818873–LC818874, MN719914, OK662943, PP739308, PX503837
Procyonidae	2	34.7	14	2	1.000 (0.500)	0.02284 (0.01142)	LC746897–LC746898
Viverridae	2	34.7	14	2	1.000 (0.500)	0.02284 (0.01142)	LC818871, LC818876
Leporidae	1	35.1	0	1	0	0	KX033489
Suidae	1	35.3	0	1	0	0	MN719912

Note: *N*, number of sequences analysed; GC%, percentage of G + C content; VS, number of variable sites; H, number of haplotypes; Hd, haplotype diversity; π, nucleotide diversity; SD, standard deviation. *Identical haplotype.

In Asian countries such as China and Japan, *T. callipaeda* demonstrates a complicated phylogeographic structure [64, 65]. This diversity may be attributed to a long evolutionary history dating back to the Middle Pleistocene, approximately 0.78 million years ago (Mya), with a 95% highest posterior density (HPD) ranging from 0.47 to 1.17 Mya, or the Late Pleistocene (0.58 Mya, 0.23–1.01 Mya) [64], during which stable populations had millennia to accrue mutations. In contrast to the high genetic heterogeneity seen in Asia, the European expansion of *T. callipaeda* represents a classic genetic bottleneck. Since its initial report in Italy in 1989 [50], the parasite has spread across nearly the entire European continent, while maintaining an almost identical genetic profile [9, 39, 41]. While H1 remains the predominant lineage in Europe, recent molecular screening has begun to detect new variants in human cases in Italy (*i.e*. H22) [9], signalling either a new introduction event or localized mutations. This paucity of diversity suggests that the European isolates originated from a single or at least two introduction events, where a very small number of parasites from a specific Asian subpopulation successfully adapted to the European vectors, such as *P. variegata* [37]. As occurred in Europe, the recent introduction of haplotype H1 into the northeast USA is probably related to an importation event from Europe [29, 53, 54].

Furthermore, the diversity of *T. callipaeda* varies across the nine host groups analysed (Table 1). Sequences detected in humans and Canidae hosts were the most numerous, showing 14 (Hd = 0.897) and 9 (Hd = 0.693) haplotypes, respectively. Substantial diversity was likewise observed in various wildlife hosts, including Ursidae (Hd = 0.800), Mustelidae (Hd = 0.733), and Felidae (Hd = 0.714). Although parasites in Procyonidae and Viverridae reached a maximum haplotype diversity of 1, these values were based on a restricted sample size of two sequences each [56, 57]. Interestingly, the high genetic diversity observed across a broad spectrum of mammalian hosts, ranging from humans and canids to various wildlife families, underscores the remarkable host plasticity and euryxenous nature of *T. callipaeda* [5, 40, 41, 56, 64]. Thus, the ability of this parasite to infect a wide array of varied hosts facilitates its environmental persistence and complicates public health interventions, since the migration of wild hosts can easily transfer “new” genetic variants into human-populated areas.

To this point, *T. callipaeda* may be a useful model for understanding how a parasite can adapt and colonise new geographical regions, despite limited genetic resources. The high degree of sequence conservation in European isolates facilitates easier molecular diagnosis, whereas the significant diversity in Asian populations requires more robust primer design or diagnostic methods to capture the entire range of circulating haplotypes [64]. The presence of multiple haplotypes in Asian endemic regions may lead to variations in pathogenicity and virulence or host specificity [30]. Meanwhile, in Europe and the USA, the uniformity of H1 haplotype has enabled rapid, predictable transmission across domestic and wild animal reservoirs, serving as a consistent source of infection for humans. Thus, genetic profiling of *T. callipaeda* is key for tracking the movement of the parasite across borders and for developing effective control strategies in both ancestral and newly invaded territories [40].

## Pathogenesis

The pathogenesis of *T. callipaeda* arises from a combination of mechanical irritation, host immune responses, and secondary opportunistic infections associated with the parasite’s activity on the ocular surface [3, 42–45]. Although *T. callipaeda* does not penetrate deeper into ocular tissues, its presence initiates a cascade of inflammation and tissue disruption that accounts for the spectrum of clinical signs [14, 45].

Once infective L3 larvae are deposited by *Phortica* flies onto the conjunctiva, they develop into adults that reside in the conjunctival sacs, third eyelids, and nasolacrimal ducts. The worm’s striated cuticle, along with buccal structures, abrades the corneal and conjunctival epithelium [3, 31, 45]. This continual mechanical irritation disrupts epithelial integrity, creating conditions that facilitate parasite persistence and reproduction.

Both larval and adult *T. callipaeda* contribute to the development of ocular thelaziosis, which typically presents with blepharospasm, ocular discharge, and conjunctivitis [3, 14, 45]. Clinical signs may range from mild, such as tearing, conjunctivitis, and keratitis, to more severe outcomes, including corneal ulcers and blindness, depending on the parasitic burden and the individual susceptibility of the host [3, 32, 48].

Mechanical damage combined with disrupted epithelial barriers facilitates secondary bacterial infections, which substantially increase disease severity [3, 6, 7, 30, 48].

Disease severity varies according to worm burden, host species, immune status, and infection duration. Dogs often exhibit mild or even no signs, whereas infection is usually asymptomatic in wild hosts [5].

Humans typically experience acute discomfort even with low parasite loads [7]. As in other mammals, in humans, infection with *T. callipaeda* produces a range of clinical manifestations, largely attributable to mechanical injury of the conjunctiva and corneal epithelium caused by the serrated keratinized surface of the worm [7, 6, 13, 21, 30, 32, 34, 39, 42, 48, 59, 61]. Rare cases of intra-ocular penetration of the worms have been documented in Thailand and other Asia countries [6, 42, 48]. The immune response of the host to parasitic secretions and excretions further exacerbates these effects. Patients commonly report ocular discomfort, such as a foreign body sensation, tearing, itching, and visual disturbances [7, 6, 13, 21, 26, 30, 32, 34, 39, 42, 48, 59, 61]. The severity of these symptoms varies among individuals.

## One Health aspect

*Thelazia callipaeda* exemplifies a One Health pathogen, as its life cycle and transmission dynamics rely on the interconnected health of humans, domestic animals, wildlife, vectors, and the surrounding environment. The parasite circulates within a complex eco-epidemiological system: dogs and wild carnivores act as reservoirs, *Phortica* fruit flies serve as biological vectors [5, 10, 39, 42]. Humans serve as accidental hosts when infected flies deposit larvae onto the ocular surface during exposure [5, 10, 42]. This interplay highlights how environmental change, vector ecology, and animal–human interactions collectively shape disease risk.

Dogs represent the principal domestic reservoir, that sustain transmission in peri-urban and rural settings [14]. Whereas wildlife (particularly foxes and wolves) function as long-term sylvatic reservoirs, maintaining the parasite independently of domestic populations and perpetuating transmission cycles even in regions with strong veterinary oversight [5, 39, 40]. This reservoir dynamic underscores the direct link between animal health and zoonotic spillover risk.

Its persistence cannot be controlled by treating humans alone but requires integrated surveillance of animal populations, vector ecology, and environmental conditions [14]. Climate and environmental change (such as warmer temperatures, forested habitats, and decaying fruit) enhance vector survival and activity, driving parasite expansion from Asia into Europe and enabling year-round transmission in tropical regions like Thailand, where the Asian vector *P. okadai* thrives in warm, humid conditions [20, 24, 34, 40, 49]. Human infection is closely linked to contact with dogs and cats, outdoor lifestyles, residence in vector-rich areas such as orchards and forests, or fruit farming activities. Children, elderly individuals, and agricultural workers are particularly vulnerable, while underdiagnosis remains common due to symptom overlap with conjunctivitis and limited clinician awareness [7, 42]. Molecular evidence shows that Southeast Asian isolates, including Thai strains, form a genetically diverse population distinct from the single founder haplotype in Europe, reflecting long-term endemicity and the role of wildlife reservoirs and vector adaptation in sustaining transmission [9, 12, 21, 64, 65]. Effective control therefore requires a coordinated One Health strategy combining routine prophylaxis and treatment of dogs and cats, wildlife monitoring, ecological research on *Phortica* flies, molecular surveillance, and community education to reduce exposure and promote early diagnosis [19, 31]. Without collaboration between veterinary medicine, human healthcare, and public health authorities, the parasite will continue to circulate silently among animals and pose an ongoing zoonotic risk [39, 42, 56].

## Treatment and control

**Figure 4:**
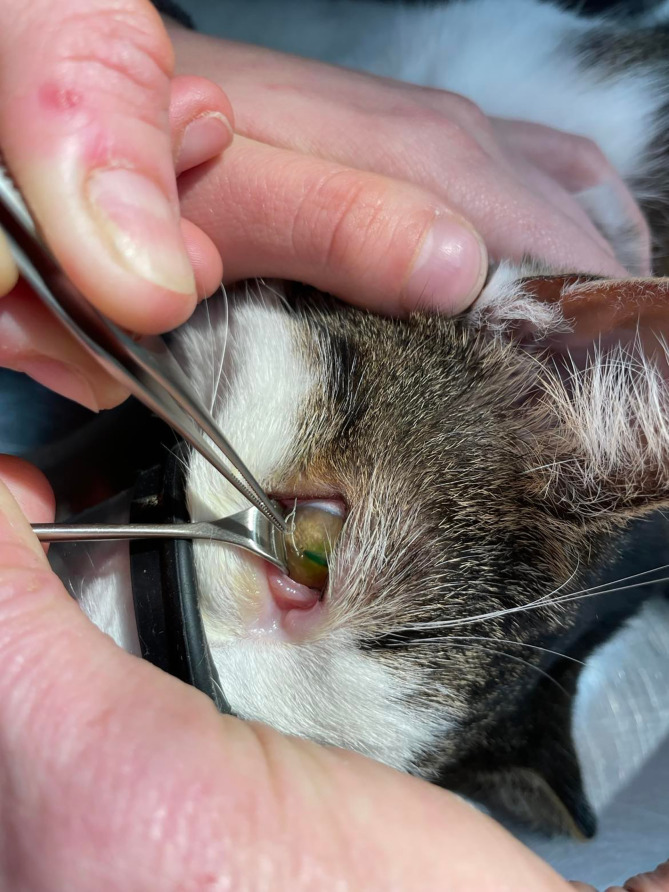
Worm extraction from a cat clinical case. Lyon, France, 2023.

Mechanical removal of worms from the conjunctival sac using forceps under topical anaesthesia, typically with oxybuprocaine hydrochloride or tetracaine hydrochloride, serves as the first line and most urgent intervention for *T. callipaeda* infection (Fig. 4) [1, 3, 45]. This technique quickly alleviates irritation and protects the cornea from further injury and is commonly followed in animals by the administration of macrocyclic lactones, applied as spot-on treatments or oral formulations to ensure complete eradication [11, 27, 38]. Before the commercialisation of active and safe macrocyclic lactone formulations in dogs, off-label subcutaneous administration of ivermectin was also performed [31, 38]. The regular use of macrocyclic lactones, including moxidectin and milbemycin oxime in dogs and moxidectin and eprinomectin in cats acts as a preventative measure, avoiding any establishment of new *Thelazia* adults [11, 27, 38]. One field study assessed the possibility to use a repellent collar based on flumethrin to prevent the transmission of *T. callipaeda* by *Phortica* flies. Unfortunately, 33% of the dogs with the collar were infected compared to no dogs treated monthly with a topical formulation of moxidectin [28]. Secondary bacterial infections are addressed with topical antibiotics when necessary [1, 58, 63].

The rising occurrence of vector-borne helminths in Europe is likely driven by the expansion of multiple arthropod vector species and underscores the need to implement measures aimed at environmental vector control and safeguarding animals at both individual and population scales [17, 19, 39]. Effective strategies for controlling vector *P. variegata* flies remain limited and insufficiently developed [17, 19, 28]. Their management is particularly difficult since these diurnal insects inhabit protected areas such as natural parks, rustic green spaces, and fruit production places, where large-scale interventions are impractical and the application of biocides carries substantial ecological risks in biodiversity-rich environments [17, 19]. Furthermore, González *et al.* (2025) [20] recently suggested the use of attractant-baited traps as a tool for both vector monitoring (entomological surveillance) and population suppression (mass capture); however, their capacity to alleviate nuisance levels has yet to be evaluated [17]. Although complementary approaches such as insecticide treatments or toxic baits can reduce nuisance levels, their application in natural habitats like forests or fruit production farms carries substantial risks for non-target organisms and threatens local biodiversity.

Identifying ocular signs facilitates ophthalmic diagnoses, since the eye is readily accessible for examination [1], monitoring wildlife such as foxes and wolves [5] and reporting expansion of the parasite helps predict risks for domestic animals and humans. However, the absence of the parasite in samples is not unexpected, as natural infection rates are exceedingly low; indeed, long term surveys have reported *T. callipaeda* larval stages in only 1.34% of fruit fly males [19, 20].

## Conclusion

In conclusion, *T. callipaeda* has emerged as a parasitic nematode of growing veterinary and public health importance, characterised by its expanding geographic distribution, broad host range, and zoonotic potential. Advances in molecular epidemiology have substantially improved understanding of the population structure and transmission dynamics of *T. callipaeda*. The contrast between the single dominant haplotype circulating in Europe and the high genetic diversity observed in Asian populations highlights distinct evolutionary histories and underscores the value of molecular tools for tracking parasite spread. From a One Health perspective, *T. callipaeda* highlights the close interconnection between animal, human, and environmental health.
